# Design of an Ultra-Wideband Transparent Wave Absorber

**DOI:** 10.3390/ma16175962

**Published:** 2023-08-31

**Authors:** Huijuan Dai, Shuying Li, Peng Dong, Yanqin Ma

**Affiliations:** 1 College of Electrical Engineering, Nanjing Vocational University of Industry Technology, Nanjing 210023, China; 2 College of Electronic and Information Engineering, Nanjing University of Aeronautics and Astronautics, Nanjing 210023, China

**Keywords:** ultra-wideband, high efficiency wave absorption, transmissibility, new composite materials

## Abstract

In this paper, a multilayer ultra-wideband transparent metamaterial wave absorber is proposed, which has the characteristics of ultra-wideband wave absorption, light transmission and flexible bending; in addition, due to the complete symmetry of the structure, the absorber has polarization insensitivity to incident electromagnetic waves. Both simulation and experimental results show that the frequency range of the microwave absorption rate is higher than 90% between 8.7 GHz and 38.9 GHz (between which most of the absorption rate can reach more than 95%), the total bandwidth is 30.2 GHz, and the relative bandwidth is 126.9%, realizing microwave broadband absorption and covering commonly used communication frequency bands such as X-band, Ku-band, and K-band. A sample was processed and tested. The test results are in good agreement with the results of the theoretical analysis, which proves the correctness of the theoretical analysis. In addition, through the selection and oxidation of indium tin (ITO) materials, the metamaterial also has the characteristics of optical transparency and flexibility, so it has potential application value in the window radar stealth and conformal radar stealth of weapons and equipment.

## 1. Introduction

Metamaterial is a kind of new artificial composite material, which is composed of the periodic or aperiodic arrangement of sub-wavelength unit structures that do not exist in nature. Because metamaterials can provide powerful electromagnetic wave regulation capabilities, they have attracted extensive attention and in-depth research from researchers in the past decade and have been widely used in beam regulation, wave absorption, information communication, and other fields. Metasurface is a two-dimensional concept of metamaterial based on the generalized Snell’s law, which is an ultra-thin two-dimensional array plane regulated by electromagnetic beams, such as wavefront phase, amplitude, and polarization.

After the rapid development of metasurfaces in recent years, a variety of novel structures have emerged involving a wide range of frequency bands, not only in the microwave band, but also including infrared and visible terahertz. With the rapid development of communication technology, multi-band and narrowband wave absorbers can no longer meet the needs of practical engineering applications. Therefore, expanding broadband to achieve ultra-wideband wave absorption has a broader application prospect.

In the field of radar communication technology, electromagnetic waves are generally divided into L-band (1 GHz–2 GHz), S-band (2 GHz–4 GHz), C-band (4 GHz–8 GHz), X-band (8 GHz–12 GHz), Ku-band (12 GHz–18 GHz), K-band (18 GHz–27 GHz), etc. The commonly used communication bands are X, Ku, and K-band, so how to realize radar stealth in these bands is very important in electromagnetic stealth. In this paper, the metasurface-absorbing structure was coated on ITO film, thus achieving the dual effects of absorbing wave and transmitting light in the X, Ku, and K bands. It has important application value in anti-radar stealth technology. For example, the cockpit of an aircraft or the porthole part of a warship must be visible while radar stealth must be realized. For this, transparent ITO film and PMMA (poly methyl methacrylate) organic glass can be used; a metasurface structure with a good wave-absorbing performance is coated on its surface.

In 2008, Landy et al. first designed a metamaterial wave absorber [1], which achieved perfect absorption of up to 99% at 11.48 GHz based on the principle of electromagnetic resonance. The metamaterial wave absorber caused a research boom. However, this absorption is based on the resonance principle and the bandwidth is usually very narrow, which does not meet the application requirements of broadband absorption. In order to expand the bandwidth, researchers have carried out a substantial amount of work, including multimode resonant absorption, loaded resistive elements, loaded active devices, composite materials technology, etc. [2,3,4,5,6,7,8,9,10,11,12,13,14,15,16]. Replacing traditional metal elements with resistive elements can expand the bandwidth either by using resistive materials, such as resistive graphite, indium tin oxide, etc., or by loading lumped resistors [6,7,8,9,10]. Loading active devices, such as varactor diodes and transistors, can adjust resonance frequency and absorb peak value, etc., thus expanding bandwidth [10,11,12,13]. Using magnetic materials such as dielectric substrates or designing periodic units such as helical and chiral structures of magnetic materials, this kind of composite material technology has the advantages of reducing overall thickness and improving large-angle incident ability while increasing bandwidth [14,15,16]. However, the above methods also have some problems, such as the complex and high cost of loading lumped resistors or active devices.

At present, most military or civil applications need a microwave absorber with a broadband absorption capability in the microwave band. In general, a broadband microwave absorber, as a microwave device that can work in multiple bands, is still the focus and difficulty of current research on microwave absorbers. The metamaterial absorber designed in this paper can not only achieve broadband microwave absorption, but also has a simple structure, convenient preparation, and low cost. Firstly, based on impedance matching theory, the impedance matching curve of the absorbing layer was deduced, which provided theoretical guidance for the optimization design of the broadband absorption of metamaterials. Secondly, a reflective metamaterial was designed, which not only had an ultra-wideband absorption performance, but was also insensitive to polarization and had the characteristics of transparency and flexibility. Finally, a sample of the designed metasurface was prepared, and its broadband absorption was verified by experiments.

## 2. Materials and Methods

**Figure 1:**
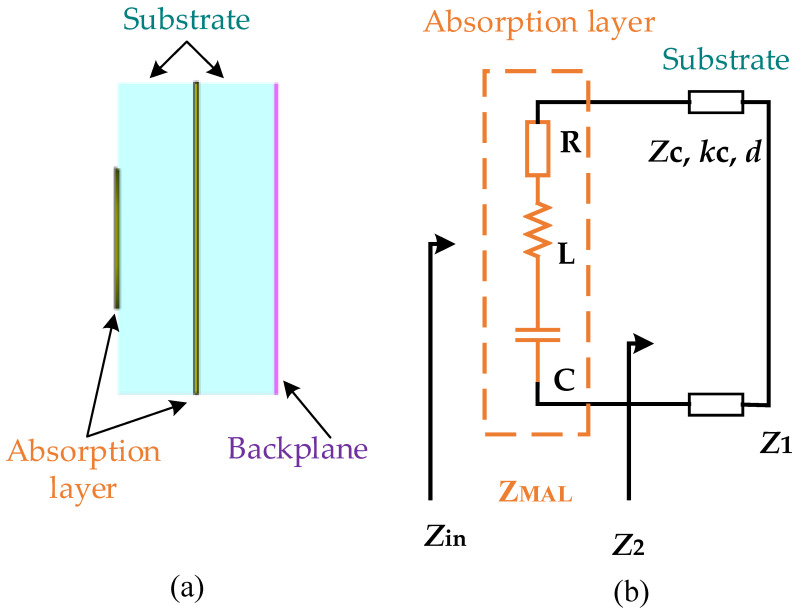
A typical three-layer reflective metasurface structure and its equivalent circuit diagram (**a**) side view; (**b**) equivalent circuit.

Figure 1a describes the case where the designed ultra-thin optical transparent metamaterial absorber is covered on the satellite solar panel. A(ω) is the absorbance of the transparent metamaterial absorber. It can be calculated by the following formula, and the reflection coefficient and transmission coefficient are expressed as R(ω) and T(ω): A(ω) = 1 − R (ω) − T(ω)= 1 − |S_11_|^2^ − |S_21_|^2^. The bottom ITO layer is approximately an ideal conductor (PEC). According to the research results, the transmission effect of 5–10 Ω ITO thin films on electromagnetic waves is similar to that of metals. In this paper, a resistance value of 5 Ω ITO thin films could achieve total reflection.

The incident electromagnetic wave is almost completely reflected in the bottom ITO layer. The bottom ITO layer is approximately an ideal electric conductor (PEC). Therefore, the absorption rate can be simplified as A(ω)= 1 − |S_11_|^2^.

The impedance matching theory is used to optimize the design of the metasurface wave absorber. The side view of the reflective metasurface unit of a typical “sandwich” structure is shown in Figure 1a. There are three layers from left to right, which are periodic unit absorbing layer, dielectric substrate, and reflective substrate. According to the transmission line theory, the structure can be equivalent to a transmission line with a short circuit at the terminal. Combined with the equivalent circuit theory, the metamaterial equivalent circuit is shown in Figure 1b, in which the resistive periodic cell-absorbing layer is equivalent to the RLC series circuit. The reflection base plate impedance is *Z*_1_ = 0. According to the transmission line theory [17,18], the input impedance of the dielectric base plate is

*Z*_2_ = j*Z*_c_tan(*k*_c_), (1)

where, *Z*_c_, *k*_c_, and *d* are, respectively, the characteristic impedance, propagation constant and thickness of the dielectric substrate, and subscript *c* represents the dielectric substrate.

The input impedance of the entire metamaterial wave absorber is

(2) $$\frac{1}{Z_{\mathrm{in}}}=\frac{1}{Z_{2}}+\frac{1}{Z_{M}},$$

where *Z*_M_ is the impedance of the absorbing layer, and subscript M is the microwave absorption layer. For reflective metamaterials, there is no transmitted wave. Based on the impedance matching theory, when the input impedance *Z*_in_ is equal to the characteristic impedance of air *Z*_0_ (≈377 Ω), there is no reflected wave. At that point, all incident electromagnetic waves enter the metamaterial and become absorbed. Hence, to achieve broadband absorption, it is necessary to match the input impedance *Z*_in_ with the characteristic impedance *Z*_0_ of the air in the widest possible frequency range. As the metamaterial shown in Figure 1a indicates, according to Formula (1) and Formula (2), when the electromagnetic parameters and thickness of the dielectric plate are defined, the input impedance *Z*_in_ is only related to the impedance *Z*_M_ of the absorbing layer. Once the dielectric plate is determined, the key to impedance matching is the design of the absorbing layer. Therefore, the design idea of broadband absorption can be further transformed into matching the actual impedance of the absorption layer with the ideal impedance in the widest possible frequency range. The so-called ideal impedance of the absorbing layer refers to the impedance value of the absorbing layer when the input impedance *Z*_in_ is equal to *Z*_0_ (the characteristic impedance of air). Therefore,

*Z*_in_ = *Z*_0_, (3)

it is deduced that the ideal impedance of the absorbing layer is [17,18]

(4) $$\frac{1}{Z_{M}^{i}}=\frac{1}{Z_{0}}-\frac{1}{Z_{2}}$$

According to Formulas (1)–(3), the ideal impedance of the absorbing layer is

(5) $$Z_{M}^{i}=\frac{jZ_{c}\tan(k_{c}d)Z_{0}}{jZ_{c}\tan(k_{c}d)-Z_{0}},$$

The real part $Z_{\mathrm{re}}^{i}$ and imaginary part $Z_{im}^{i}$ of the ideal impedance of the absorbing layer can be extracted by MATLAB as

(6) $$Z_{re}^{i}=re(Z_{M}^{i}),$$

(7) $$Z_{im}^{i}=\mathrm{im}(Z_{M}^{i})$$

The actual impedance curve of the absorbing layer can be inversely calculated from the reflection coefficient simulated by the software. By definition, the reflection coefficient is

(8) $$S_{11}=\frac{Z_{\mathrm{in}}-Z_{0}}{Z_{\mathrm{in}}+Z_{0}},$$

then the actual input impedance of metamaterial is

(9) $$Z_{\mathrm{in}}^{a}=Z_{0}\frac{1+S_{11}}{1-S_{11}},$$

and the actual impedance of the absorbing layer is

(10) $$Z_{M}^{a}=\frac{Z_{2}Z_{in}^{a}}{Z_{2}-Z_{in}^{a}}=\frac{Z_{2}Z_{0}(1+S_{11})}{Z_{2}-Z_{0}-S_{11}(Z_{2}+Z_{0})}$$

The real part $Z_{re}^{a}$ and imaginary part $Z_{im}^{a}$ of the actual impedance of the absorbing layer are also extracted by MATLAB:

(11) $$Z_{re}^{a}=\mathrm{re}(Z_{M}^{a}),$$

(12) $$Z_{im}^{a}=\mathrm{im}(Z_{M}^{a})$$

Using MATLAB to conduct a series of optimization analysis, the broadband absorption impedance matching curve shown is finally obtained. When the actual impedance of the real part of the absorbing layer is around 155 Ω and the resonance point of the actual impedance imaginary part and the ideal impedance imaginary part is close to each other, broadband microwave absorption can be achieved.

## 3. Structural Design and Simulation Optimization

**Figure 2:**
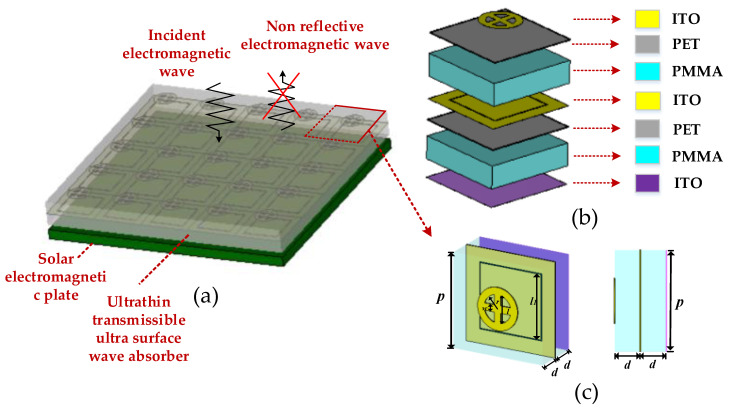
(**a**) Schematic diagram of a satellite solar panel covering the broadband transmissible metasurface absorber; (**b**) unit structure distribution diagram of the transparent metamaterials absorber; (**c**) 3D view and side view of the absorbing metasurface unit.

For ultra-wideband characteristics, we have designed an ultra-wideband absorber based on a multilayer patterned resistance film, and its overall unit structure is shown in Figure 2b. The figures of the two layers of resistance film with the same square resistance is, respectively, shown in Figure 2b. They are seamlessly placed on PET material with a dielectric constant of 3 and a thickness of 0.1 mm and are stacked with PMMA plates with the same thickness (dielectric constant of 2.25 and thickness of 2 mm). The first layer of resistance film is composed of a circle with an inner and outer radius of *r*_1_ = 2.5 mm and *r*_2_ = 3.75 mm (*r*_1_ = 1.5 *r*_2_) and a cross in the middle. The pattern of the second layer of resistance film is a gap groove with a width of 0.1 mm etched in the middle of the entire ITO film. The length of the inner side of the gap groove is *l*_1_ = 6.8 mm, and the nearest distance from the periphery of the gap to the four sides of the cell is 1.5 mm; the bottom is the metal back plate. The square resistance of the entire ITO film is 5 Ω. The absorber utilizes the principle of multi-layer interference cancellation and the corresponding ohmic loss to achieve efficient broadband absorption.

The broadband transmissible metamaterial absorber unit has a five-layer structure, as shown in Figure 2b above. The first layer is an absorbing structure for processing and etching ITO. The dielectric constant of ITO material is 3, and the unit circumference is *p* = 10 mm. It is composed of a circle with internal and external radii of *r*_1_ = 2.5 mm, *r*_2_ = 3.75 mm, and a cross in the middle. Its square resistance value is 155 Ω, which is etched on PET film, and the thickness is ignored; the second layer is PMMA material, with thickness of *d* = 2 mm and a dielectric constant of 2.25. The third layer and the first layer are completely the same as PET material, and the fourth layer and the second layer are completely the same as PMMA material, with a thickness of *d* = 2 mm. The fifth layer is the entire ITO film with a square resistance of 5 Ω. The five-layer structure together forms a whole wave absorbing unit, and meets the requirements of *l*_2_ = 2 × *r*_2_, *w*_2_ = *r*_2_/2, *r*_1_ = 3 × *r*_2_/2, *w*_1_ = 6.8 mm, *w*_0_ = 0.1 mm.

**Figure 3:**
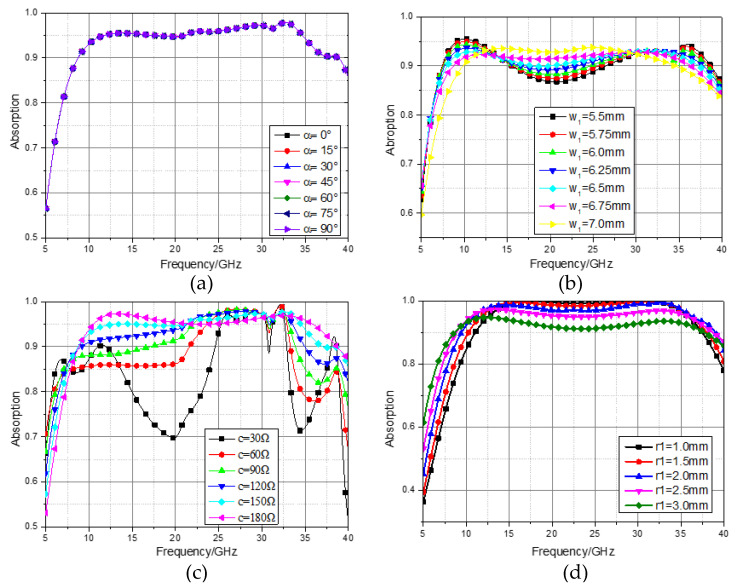
Effect of different parameter sizes on absorption. (**a**) Absorbance at different polarization angles; (**b**) absorbance at different cross lengths; (**c**) absorbance of the first ITO film with different resistance values; (**d**) absorbance of different ring outer diameter *r*_2_.

Since each layer of resistance film is seamlessly covered on PET media with a thickness of 0.1 mm, the overall thickness of the wave absorber is 4.2 mm. The stability of the polarization angle and incident angle is also a major criterion for evaluating the performance of the absorber. The influence of the change in polarization angle on the absorption rate is shown in Figure 3a. It can be seen that when the polarization angle changes from 0° to 90°, the absorption performance of the incident electromagnetic wave of the absorber still remains unchanged. This is because each layer of the absorber has complete symmetry in the periodic structure, so the absorber has good polarization angle insensitivity, that is, the change in the polarization angle of the electromagnetic wave hardly affects the absorption rate; the wave absorber can absorb the incident electromagnetic wave in any polarization direction.

The influence of the change in the middle cross length *w*_1_ on the absorption rate is shown in Figure 3b. It can be seen that with the change in the value of *w*_1_ from 5.5 mm to 7.0 mm, the bandwidth of the absorption rate will gradually narrow, but the average absorption rate in the band will increase. Therefore, when selecting the value of *w*_1_, the absorption bandwidth and absorption rate should be comprehensively considered. In this paper, the selection of *w*_1_ = 6.8 mm is in combination with this aspect. The absorption of the first layer of ITO film when the resistance value changes is shown in Figure 3c. As the value of *c* changes from 30 Ω to 180 Ω, the absorption gradually increases. When *c* = 155 Ω, the absorption and absorption bandwidth are relatively optimal.

## 4. Performance Analysis

**Figure 4:**
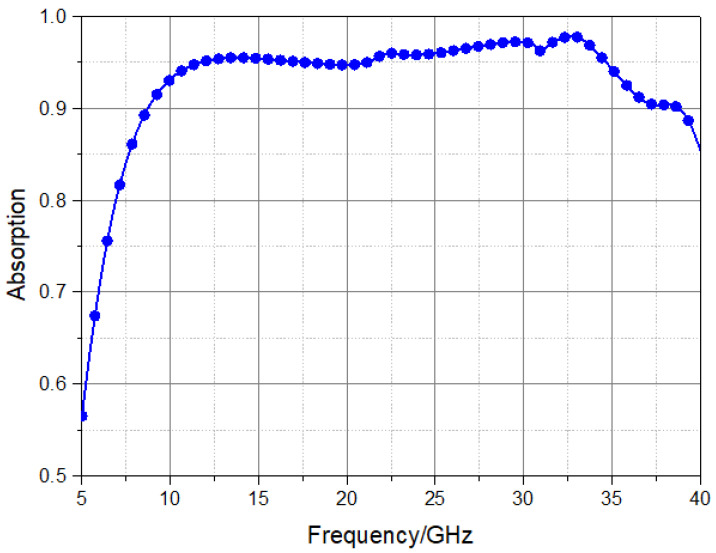
Absorption curve of the designed transmissible wave absorber.

Under normal electromagnetic wave incidents, the transmissible metamaterial absorber shows an absorption rate of more than 90% at any polarization in the 8.7 GHz–38.9 GHz frequency band, as shown in Figure 4 below. When the electromagnetic wave is obliquely incident, the transparent metamaterial absorber sample with a thickness of 4.2 mm is processed and manufactured. Simulation by CST shows that the designed transparent metamaterial absorber achieves full coverage of the absorption of the X band, K_u_ band, K band, and K_a_ band and basically achieves more than 95% of the electromagnetic wave absorption in these bands while achieving effective light transmission.

**Figure 5:**
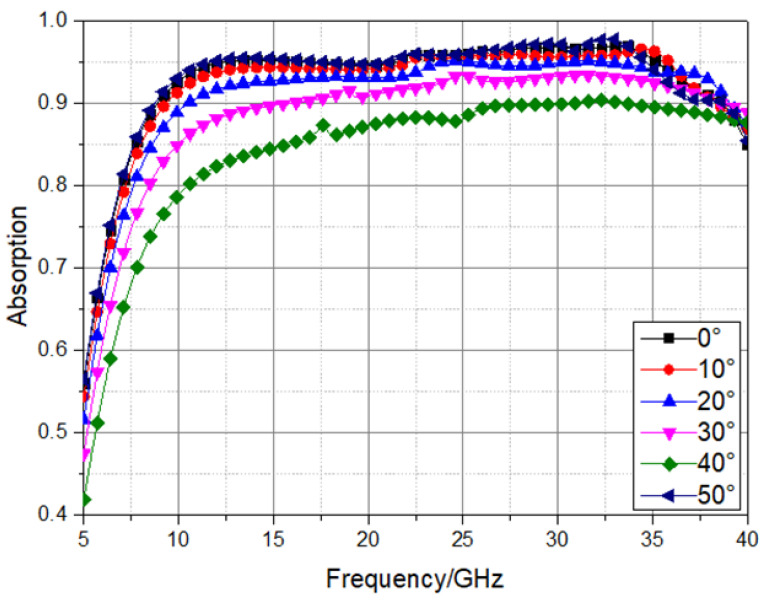
Ultra-wide band-absorbing performance at different incident angles.

Next, we explore the absorption rate of the absorber at different incident angles, as shown in Figure 5. In TE mode, when the incident angle changes from 0° to 50°, the absorption rate decreases as a whole. This is because changes of the incident angle result in change in the overall wave impedance of the absorber corresponding to the TE mode, which does not match the free space wave impedance, leading to a decrease in the absorption rate. In TM mode, when the incident angle changes from 0° to 50°, frequency deviation occurs, which is about 1 GHz. At this time, the stability of the incident angle is not satisfied. This is because when the incident angle increases, the electric length of the projection parallel to the electric field decreases, resulting in an increase in the resonance frequency, which leads to frequency deviation.

**Figure 6:**
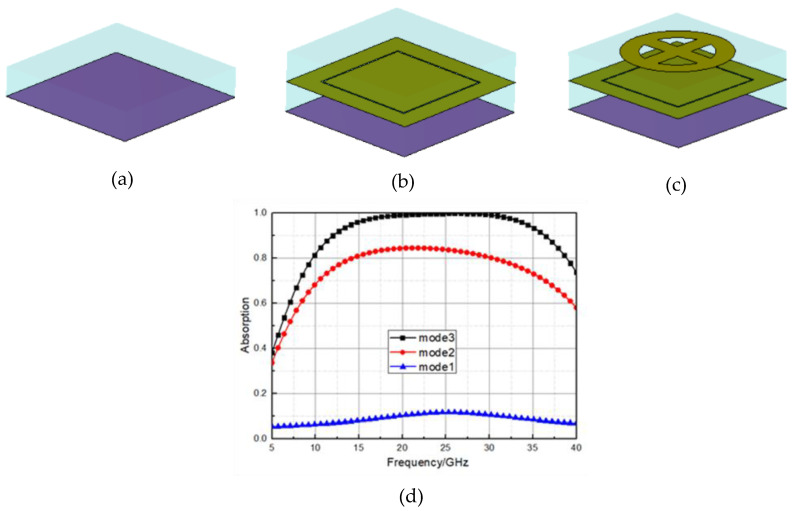
Effect of different resistance layers on wave absorbers: (**a**) the bottom resistance film with the dielectric layer; (**b**) removal of only the first conductive pattern; (**c**) complete absorbing unit; (**d**) comparison of absorption rates of the three modes.

In order to explore the ultra-wideband absorption mechanism of the proposed microwave absorber based on the multilayer patterned resistance film, we mainly study the contribution of each layer of resistance film to the absorption. Figure 6 shows the different combination spectra based on different patterned resistance film layers. It can be seen that the bottom resistance film provides low electromagnetic wave absorption effect in the low frequency region, mainly providing wave absorption in the 20 GHz–30 GHz frequency band. The second layer of resistance film mainly increases the bandwidth to 15 GHz and shifts the absorption peak provided by the bottom layer of resistance film to a low frequency; the addition of the third layer of ring and cross pattern resistance film expands the absorption frequency band to more than 30 GHz and greatly improves the absorption rate of this frequency band close to perfect absorption between 20 GHz and 30 GHz. The absorption bandwidth can be effectively extended to 30 GHz and the low frequency can be extended to 10 GHz by introducing the top opening combined toroidal resistance film into the ultra-wideband wave absorber, while the high-performance absorption rate is also ensured.

**Figure 7:**
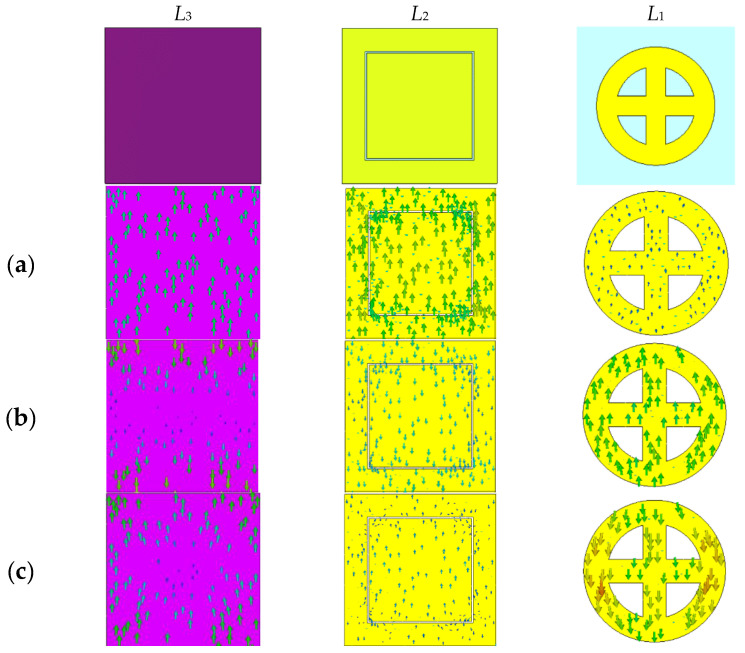
Surface current simulation results of different layers at different frequency points (**a**) 12 GHz; (**b**) 22 GHz; (**c**) 32.8 GHz.

In addition, the coupling of conductive patterns between different layers further improves the absorption rate in the entire absorption band. We selected three frequency points of 12 GHz, 22 GHz, and 32.8 GHz and described the surface current distribution on different resistance film layers under these absorption peak frequency points in order to further explore the absorption principle of the ultra-wideband wave absorber. As shown in Figure 7a, for the absorption peak at 12 GHz, the surface current is concentrated on the bottom resistance film *L*_3_, because the bottom resistance film plays an important role in the dissipation of electromagnetic waves. Moreover, the flow direction of the surface current on the ground plate is antiparallel to the flow direction of the surface current on the bottom *L*_3_, which excites the magnetic dipole resonance, leading to strong wave absorption. As shown in Figure 7b, the absorption at 22 GHz is mainly caused by the coupling between the resistance layers. There is a reverse flow of surface current between *L*_1_, *L*_3_ layers, and intermediate layers *L*_2_ and *L*_3_. The magnetic resonance is excited by the reverse parallel flow direction of current on these layers. As shown in Figure 7c, at the resonance peak of 32.8 GHz, the surface current distribution is mainly concentrated on the top *L*_1_ layer. Therefore, the power loss on the top *L*_1_ layer is the reason for strong absorption at this resonant frequency. At the same time, the surface current on the top *L*_1_ layer is antiparallel to the surface current on the middle layer *L*_2_, which excites magnetic resonance, and the coupling between the resistance films of each layer causes high absorption at 32.8 GHz.

Therefore, the reason why the absorber can have an ultra-wideband working frequency band and an efficient electromagnetic wave absorption rate is that each individual resistance film layer provides resonance absorption of different frequency bands, which can be combined to expand the broadband, and the coupling between multiple conductive pattern layers can effectively improve the absorption rate.

## 5. Experimental Verification

**Figure 8:**
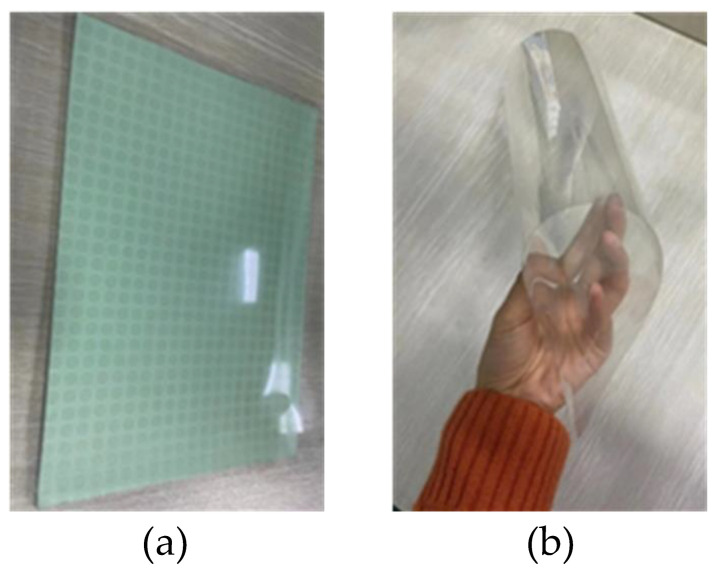
Schematic diagram of processed samples: (**a**) the absorbing material shows high transparency; (**b**) The absorbing material has good flexibility.

To verify the correctness of the above simulation results, a 300 mm × 300 mm sample was processed and manufactured. As shown in Figure 8, the first and third layers of ITO with 155 Ω were etched on the PET conductive film, the second and fourth layers were both made of a 2 mm thick medium plate, and the fifth layer was an ITO film with a resistance of 5 Ω. The absorbing layer, the dielectric substrate, and the reflecting substrate were pasted together by optical transparent glue. The photos of the final prepared samples are shown in Figure 8. Figure 8a is the photo of the sample placed on the book, which shows that the metamaterial has high optical transparency characteristics. Figure 8b is the photo of the sample in a bending state, which proves that it has good flexibility and can be conformal with the surface of the weapon equipment.

**Figure 9:**
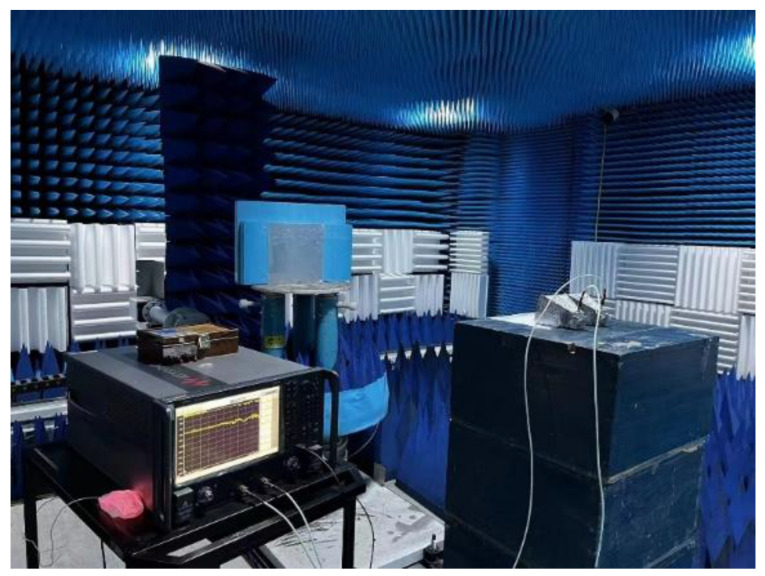
Schematic diagram of experimental test.

**Figure 10:**
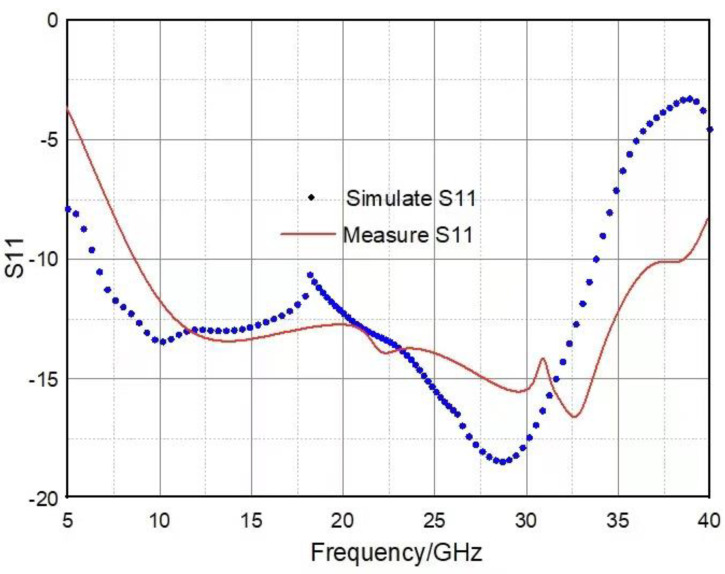
Schematic diagram of the comparison between experimental test results and simulation results.

The experimental test was carried out in a microwave anechoic chamber, as shown in Figure 9. The main measuring equipment was a vector network analyzer (Agilent, Santa Clara, CA, USA, N5245A) and three pairs of identical horn antennas (because the tested frequency band was ultra-wideband, which was divided into three sections to test three bands of 2 GHz–18 GHz, 18 GHz–26.5 GHz, and 26.5 GHz–40 GHz). The comparison between the measured reflection coefficient versus frequency curve and the simulation results is shown in Figure 10. It can be seen that the simulation results are consistent with the test results whether the TE wave or TM wave is incident vertically, which verifies the broadband microwave absorption performance of the metamaterial. According to Figure 10, when the electromagnetic wave is incident vertically, the frequency range with the reflection coefficient less than −10 dB measured in the experiment is 8.7 GHz–38.9 GHz, the total bandwidth is 30.2 GHz, and the relative bandwidth is 91.4%.

Table 1 lists the performance comparison of the metamaterial absorber designed in this paper and other relevant references. It can be seen that the metamaterial absorber designed in this paper not only has an ultra-wide absorption band (both relative and absolute bandwidth are very wide) and thin thickness, but also has the characteristics of transparency and flexibility and has polarization insensitive characteristics. Therefore, the metamaterial absorber designed in this paper has a broad potential application value. 

## 6. Conclusions

Firstly, based on the impedance matching theory, a broadband, transparent, and flexible metamaterial absorber was designed. The simulation results show that its absorption bandwidth of more than −10 dB is 8.7 GHz–38.9 GHz, and its absorption rate of more than 95% is in the common communication bands X, K_u_, and K. Because the two layers of the pattern structure of the element were of type symmetry structure, the metasurface was insensitive to the polarization of the incident electromagnetic wave; by selecting PMMA as the dielectric substrate and ITO/PET film as the absorbing layer and reflecting substrate, the metamaterial also had high transmittance and flexible bending characteristics. Finally, a metamaterial sample was prepared, and the experimental results were consistent with the simulation results, which verified the correctness of its broadband absorbing characteristics, the correctness of the metamaterial simulation design, and the feasibility of broadband application. The designed metasurface wave absorber not only has broadband wave absorption performance, but also is insensitive to polarization, and has the characteristics of transparency, flexibility, a simple structure, and convenient preparation. This structure has the advantages of simple structure, light transmission, wide band, high absorption efficiency, and bending, which can effectively improve the working efficiency of satellite detection radar and the broadband microwave stealth of solar panels.

## Figures and Tables

**Table 1:** Comparison with the previously reported absorbing metasurfaces.

Refs.	≥90% Absorption (GHz)	≥90% Absorption (GHz)	≥90% Absorption	≥75% (TE) Absorption (≤θ)	≥80% ™ Absorption (≤θ)	Polarization Insensitivity	Optically Transparent
[19]	10.04–10.72	0.68	6.551%	50	-	Yes	No
[20]	8.81–13.83	5.02	44.35%	40	-	No	No
[21]	21–42.5	21.5	67.72%	45	30	No	Yes
[22]	11.44–20.0	8.56	54.45%	50	50	Yes	No
[23]	7.5–15.0	7.5	66.67%	50	60	No	No
[24]	8.3–17.4	9.1	70.82%	45	50	Yes	Yes
[25]	8.0–15.0	7	60.87%	50	40	Yes	No
This work	8.7–38.9	30.2	126.9%	50	50	Yes	Yes

## Data Availability

The raw/processed data and modeling codes required to reproduce these findings cannot be shared at this time, as the data also form part of an ongoing study.
